# Suicidal ideation and suicide attempts in healthcare professionals during the COVID-19 pandemic: A systematic review

**DOI:** 10.3389/fpubh.2022.1043216

**Published:** 2022-12-06

**Authors:** Juan Jesús García-Iglesias, Juan Gómez-Salgado, Francisco Javier Fernández-Carrasco, Luciano Rodríguez-Díaz, Juana María Vázquez-Lara, Blanca Prieto-Callejero, Regina Allande-Cussó

**Affiliations:** ^1^Department of Sociology, Social Work and Public Health, Faculty of Labour Sciences, University of Huelva, Huelva, Spain; ^2^Safety and Health Postgraduate Programme, Universidad Espíritu Santo, Guayaquil, Ecuador; ^3^Department of Obstetrics, Punta de Europa Hospital, Algeciras, Spain; ^4^Nursing and Physiotherapy Department, Faculty of Nursing, University of Cádiz, Algeciras, Spain; ^5^Faculty of Health Sciences, University of Granada, Ceuta, Spain; ^6^Department of Nursing, University of Huelva, Huelva, Spain; ^7^Department of Nursing, Faculty of Nursing, Podiatry and Physiotherapy, University of Seville, Seville, Spain

**Keywords:** COVID-19, health personnel, suicide, mental health, risk factors, public health

## Abstract

**Background:**

COVID-19 has caused a series of economic, social, personal, and occupational consequences that may affect the mental health of healthcare workers (HCWs), with the consequent risk of developing suicidal ideation and behaviors.

**Objectives:**

The aim of this study was to identify the main risk factors that may predispose HCWs to suicidal ideation and suicide attempts during the COVID-19 pandemic.

**Methods:**

A systematic review of studies published between January 2020 and August 2022 was conducted following the PRISMA guidelines in the following electronic databases: Pubmed, Scopus, Web of Science, CINAHL, and PsycINFO. Methodological quality was assessed using the critical appraisal tools for non-randomized studies of the Joanna Briggs Institute (JBI). The followed protocol is listed in the International Prospective Register of Systematic Reviews (PROSPERO) with code CRD42022340732.

**Results:**

A total of 34 studies were included in this review. There are a number of underlying factors such as higher rates of depression, anxiety, pre-pandemic lifetime mental disorders or previous lifetime suicide attempt, living alone, having problems with alcohol and/or other drugs, etc. that favor the emergence of suicidal tendencies and ideation in times of COVID-19. Similarly, the pandemic may have precipitated a series of factors such as economic concerns, assessing one's working conditions as poor, having family members or friends infected, changes in services or functions, and feeling discriminated against or stigmatized by society. Other factors such as age, sex, or type of healthcare worker show differences between studies.

**Conclusion:**

Organizations should ensure the adoption of strategies and programmes for early detection of suicides as well as increased attention to the mental health of professions with a high workload.

**Systematic review registration:**

PROSPERO, identifier CRD42022340732.

## Introduction

The WHO (1) estimates that there are more than 700,000 suicide deaths per year. This phenomenon is considered a complex public health problem due to its multivariate casuistry, where psychological, sociocultural, biological, economic, and personal factors may converge.

According to the Columbia Classification Algorithm of Suicide Assessment (C-CASA) (2), there are eight categories related to suicidal behavior. Among these eight categories, we find completed suicide (self-injurious behavior that triggers the death of an individual); suicide attempt (potentially self-injurious behavior in which the individual had the intention to commit suicide); preparatory acts toward imminent suicidal behavior (where the individual takes steps to self-harm but the self or third parties prevent the act of self-harm itself); suicidal ideation (passive thoughts about wanting to be dead or active thoughts about killing oneself but not accompanied by preparatory behavior); and self-injurious behavior (self-injurious behavior where the associated intention to die is unknown and cannot be inferred).

In an attempt to explain what drives a person to commit suicide, to plan and think about it, a number of theories have been established. According to the Interpersonal Theory of Suicide (3), a person will not attempt suicide unless they have both the desire to die by suicide and the ability to do so, so there must be thwarted belongingness and perceived burdensomeness. According to the Integrated Motivational-Volitional Model of Suicidal Behavior, individuals go through three phases in which feelings of defeat and entrapment are key elements, and in which the biopsychosocial context in which suicidal behavior arises, the factors involved in suicidal ideation, and those factors linked to the transition between suicidal ideation and suicidal action are precipitating factors (4). In addition, there are other theories (5) such as the Eco-developmental Model of Suicide Attempts (6), in which individual, relational, community, and social factors converge; the Cultural Theory and Model of Suicide, where there is a cultural component of suicide that goes beyond the individual and can affect relationships, the community, and society; or the Three-Step Theory (7), in which factors such as grief and hopelessness, lack of connection or attachment to other people, the absence of a meaningful job or life project coexist with the capacity for suicide itself, among others.

All these theories refer to risk or predisposing factors for suicidal behavior. The risk factor construct is understood in terms of probability and refers to a variable or factor that predisposes an individual to develop a certain disease or pathology (8). For the general population (3, 9), there are a number of risk factors that predispose to suicidal behavior such as childhood abuse, mental disorders and previous suicide attempts, situations of social isolation, despair, lack of resources, family conflict, incarceration or unemployment, problems with authorities, alcohol and other drug abuse, family history of suicide, diagnosis of physical illness, serotonergic dysfunction, seasonal variation, and personal traits such as impulsivity, predisposition to struggle, and low self-esteem or feelings of shame or guilt. Likewise, females have a higher risk of attempted suicide than males, although males have higher rates of completed suicide (10, 11), a phenomenon known as the gender paradox (12). With all these factors, we have now added a variable such as the pandemic caused by COVID-19 [Coronavirus disease 2019, an infectious disease caused by the Severe Acute Respiratory Syndrome coronavirus 2 (SARS-CoV-2)]. The COVID-19 pandemic has led to confinement, limited mobility, changes in people's social lives, and economic problems that have negatively affected both the mental health and wellbeing of individuals (13). Compared to previous epidemics (14), suicide rates may have increased during and after health crises, as corroborated by a systematic review on suicidal ideation and behaviors during the COVID-19 pandemic (15). It is true that in recent years it has become apparent that some occupations are more prone to suicide risk, such as healthcare workers (HCWs), who are 3–5 times more likely to be at risk of suicide (16) and who, as expected, have been closely affected by the different epidemic waves of the COVID-19 pandemic (17). This suggests that suicide rates may be increased by pre-existing or emerging mental health conditions. In fact, it is estimated that the suicide rate among male clinicians is almost 1.5 times higher than that of female clinicians and 2.3 times higher than in the general population (11, 18). Among female nurses, there are also higher suicide rates than in the general population (19). Healthcare professionals appear to have occupation-specific risks for suicide as a result of their highly stressful work environment or the impact of the situations they experience, such as being involved in a physician error, among others (11). The consequences of suicidal ideation can lead to suicide attempts and completed suicide with the resulting personal loss. More specifically, in clinical practice, such thoughts can affect adequate professional performance due to a lack of empathy, kindness, compassion, and active listening skills, in detriment to the quality of care provided (17).

Especially at the beginning of the pandemic, HCWs may have felt worried about infecting their loved ones, may have been afraid of the disease, felt stigmatized and isolated by society, suffered traumatic experiences and ethical dilemmas, and may have been subjected to high levels of stress, anxiety, and depression (20, 21). In addition, in the work environment, many HCWs have lacked personal protective equipment, have had increased patient load, have had to make difficult decisions, have witnessed a high number of deaths of patients under their care, have been forced to double shifts, and have been relocated from their services (22). All this factors have had the potential to undermine the mental health of HCWs, with the consequent risk of developing suicidal ideation and behavior (23). While it is true that the pandemic has fluctuated, a study in Bangladesh (15) found that, from April 2020 to July 2020, the prevalence of suicidal ideation had increased from 5 to 19%, similar figures to those reported by Mortier et al. (24), which range from 4.4 to 13%. In another study, suicidal thoughts had a prevalence of 11% among HCWs, compared to 6% in the general population (25). It should not be disregarded that suicidal ideation is a predictor of future suicide attempts and suicide deaths (26), hence the importance of addressing it at early stages. In this regard, Sahimi et al. (27) and Rodney et al. (21) found that 17% of physicians reported suicidal ideation, of whom 1% had attempted suicide. Furthermore, HCWs are more likely than non-HCWs to succeed in suicide attempts as they have greater access to more lethal drugs and have knowledge about the sufficient dosage to end their lives (28). In the systematic review by Dutheil et al. (20) carried out in 2019, ~1.0% of physicians attempted suicide and 17% of physicians had suicidal ideation. These figures decreased as the pandemic progressed, especially in European countries. The monitoring of cases throughout the pandemic could require a specific approach to this issue, and also more longitudinal studies should be carried out to assess events in this field. As can be seen, there is wide variability and contradictions between different research results affecting suicide. For this reason, the aim of this review was to identify the main risk factors that may predispose a healthcare professional to suicidal ideation and suicide attempts during the COVID-19 pandemic.

## Materials and methods

### Study design

A systematic review was conducted following the PRISMA guidelines (Preferred Reporting Items for Systematic reviews and Meta-Analyses) (29). The protocol followed is listed in the International Prospective Register of Systematic Reviews (PROSPERO) with code CRD42022340732. Ethical aspects.

### Databases and search strategy

The databases used were Pubmed, Scopus, Web of Science, Cumulative Index to Nursing and Allied Health Literature Complete (CINAHL), and PsycINFO. The search strategy used to collect the studies in the aforementioned databases was based on the key words obtained from the PECOT strategy, which yielded the research question: What factors may influence a healthcare professional to present suicide attempts and suicidal ideation during the COVID-19 pandemic? (Table 1).

**Table 1:** PECOT format: keywords.

**Population**	Healthcare professionals
**Event**	Suicidal tendencies: suicide attempts and suicidal ideation
**Comparison**	Risk/protective factors
**Outcomes**	Number of cases, risk vs. protective factors, occupational vs. non-occupational factors, quantification of suicidal tendencies, comparison of levels before vs. during the COVID-19 pandemic, comparison according to type of profession/service
**Time**	During the COVID-19 pandemic
**Research question**
What factors may influence a healthcare professional to present suicide attempts and suicidal ideation during the COVID-19 pandemic?	

Following these keywords, the Medical Subject Headings (MeSH) thesaurus was consulted, yielding the descriptors health personnel, COVID-19, and suicide. In order to expand the search for published studies in line with the subject of the study, the use of free terms together with the MeSH descriptors was put in practice through the use of the Boolean operators AND and OR (Table 2).

**Table 2:** Search terms.

**MeSH^†^ terms**	**Terms**
Health personnel	Healthcare professionals OR Healthcare workers OR Healthcare providers OR Physician* OR Nurse* OR Doctor*
COVID-19	COVID-19 OR Coronavirus OR 2019-ncov OR SARS-CoV-2 OR Cov-19 OR pandemic
Suicide	Suicide OR Suicide Attempt* OR Suicide Completed OR suicidal ideation OR suicidal behavior OR self-harm* OR self-injury

† MeSH, Medical Subject Headings.

Table 3 shows the search strategy used, carried out on 08 August 2022 for each of the above-mentioned databases during the search process.

**Table 3:** Search strategy for each database.

**Database**	**Search strategy**	**Results**
Pubmed	((((((((Suicide[Title/Abstract]) OR (Suicide Attempt*[Title/Abstract])) OR (Suicide Completed[Title/Abstract])) OR (suicidal ideation[Title/Abstract])) OR (suicidal behavior[Title/Abstract])) OR (self-harm*[Title/Abstract])) OR (self-injury[Title/Abstract])) AND ((((((Healthcare professionals[Title/Abstract]) OR (Healthcare workers[Title/Abstract])) OR (Healthcare providers[Title/Abstract])) OR (Physician*[Title/Abstract])) OR (Nurse*[Title/Abstract])) OR (Doctor*[Title/Abstract]))) AND ((((((COVID-19[Title/Abstract]) OR (Coronavirus[Title/Abstract])) OR (2019-ncov[Title/Abstract])) OR (SARS-CoV-2[Title/Abstract])) OR (Cov-19[Title/Abstract])) OR (pandemic[Title/Abstract])) Filters: from 2020 - 2022	133
Scopus	(TITLE-ABS-KEY (suicide) OR TITLE-ABS-KEY (“suicide attempt*”) OR TITLE-ABS-KEY (“suicide completed”) OR TITLE-ABS-KEY (“suicidal ideation”) OR TITLE-ABS-KEY (“suicidal behavior”) OR TITLE-ABS-KEY (self-harm*) OR TITLE-ABS-KEY (self-injury) AND TITLE-ABS-KEY (“Healthcare professionals”) OR TITLE-ABS-KEY (“Healthcare workers”) OR TITLE-ABS-KEY (“Healthcare providers”) OR TITLE-ABS-KEY (physicians) OR TITLE-ABS-KEY (nurses) OR TITLE-ABS-KEY (doctors) AND TITLE-ABS-KEY (covid-19) OR TITLE-ABS-KEY (coronavirus) OR TITLE-ABS-KEY (2019-ncov) OR TITLE-ABS-KEY (SARS-CoV-2) OR TITLE-ABS-KEY (cov-19) OR TITLE-ABS-KEY (pandemic) AND (LIMIT-TO (PUBYEAR, 2022) OR LIMIT-TO (PUBYEAR, 2021) OR LIMIT-TO (PUBYEAR, 2020)	279
Web of science	TOPIC: (Suicide OR Suicide Attempt* OR Suicide Completed OR suicidal ideation OR suicidal behavior OR self-harm* OR self-injury) AND TOPIC: (Healthcare professionals OR Healthcare workers OR Healthcare providers OR Physician* OR Nurse* OR Doctor*) AND TOPIC: (COVID-19 OR Coronavirus OR 2019-ncov OR SARS-CoV-2 OR Cov-19 OR pandemic) Refined By: Publication Years: 2022 or 2021 or 2020	240
CINAHL	AB (Suicide OR Suicide Attempt* OR Suicide Completed OR suicidal ideation OR suicidal behavior OR self-harm* OR self-injury) AND AB (Healthcare professionals OR Healthcare workers OR Healthcare providers OR Physician* OR Nurse* OR Doctor*) AND AB (COVID-19 OR Coronavirus OR 2019-ncov OR SARS-CoV-2 OR Cov-19 OR pandemic) Refined By: Publication Years: 2022 or 2021 or 2020	89
PsycInfo	tiab(Suicide OR Suicide Attempt* OR Suicide Completed OR suicidal ideation OR suicidal behavior OR self-harm* OR self-injury) AND tiab(Healthcare professionals OR Healthcare workers OR Healthcare providers OR Physician* OR Nurse* OR Doctor*) AND tiab(COVID-19 OR Coronavirus OR 2019-ncov OR SARS-CoV-2 OR Cov-19 OR pandemic) Filters: from 2020 to 2022	48
**Date of search** 08/08/2022	**Total**	789

### Selection criteria

The following inclusion criteria were used for the selection of articles: (1) design criterion: cross-sectional, longitudinal, and interventional studies; (2) language criterion: articles published in English, Spanish, French, and Portuguese; (3) data collection period: articles where data were collected during the COVID-19 pandemic and; (4) outcome measure criterion: articles measuring any of the following values/indicators: number of cases or proportion of suicidal tendencies, risk factors vs. protective factors, occupational vs. non-occupational factors, comparison of levels before vs. during the COVID-19 pandemic, comparison according to type of profession/service/level of exposure to COVID-19. On the other hand, the exclusion criteria were (1) language reasons (language other than English, Spanish, French, and Portuguese); (2) low scientific-technical quality after applying the quality assessment tool; (3) by type of article (published conference proceedings, conference abstracts, and theses or studies including animals); (4) population: students who do not perform healthcare practices; and (5) studies in which the collection date, study population, or measurement instrument could not be determined.

### Data collection and extraction

For data collection and extraction, two researchers independently searched the databases according to agreed keywords. They eliminated duplicate studies and selected articles that could be included according to the previously established criteria after reading the title and abstract. Then, these two authors reviewed the full text of the studies that could potentially be included in the review and the decision to include or exclude them in the review was made at this stage by consensus, with a third author having the discretion to include or not a study in case of discrepancy between the two authors. For the selection report, the two authors collected information from the studies regarding authorship, year of publication, country and date of data collection, overall objective, study design, population, measurement instrument, and main results; in addition, the results of the Joanna Briggs Institute (JBI) critical appraisal tool were added.

### Methodological quality assessment

Two reviewers independently determined the methodological quality of the selected studies using the critical appraisal tools for non-randomized studies of the JBI of the University of Adelaide (30). These tools allow assessing the methodological quality of a study and determining the extent to which a study had minimized the possibility of bias in its design, development, and/or analysis. The versions for cross-sectional quantitative studies (eight items) and for case control studies (10 items) were used, setting the cut-off point at 6/8 for the former and 8/10 for the latter to accept their inclusion in this review (Supplementary material).

## Results

### Literature search

**Figure 1:**
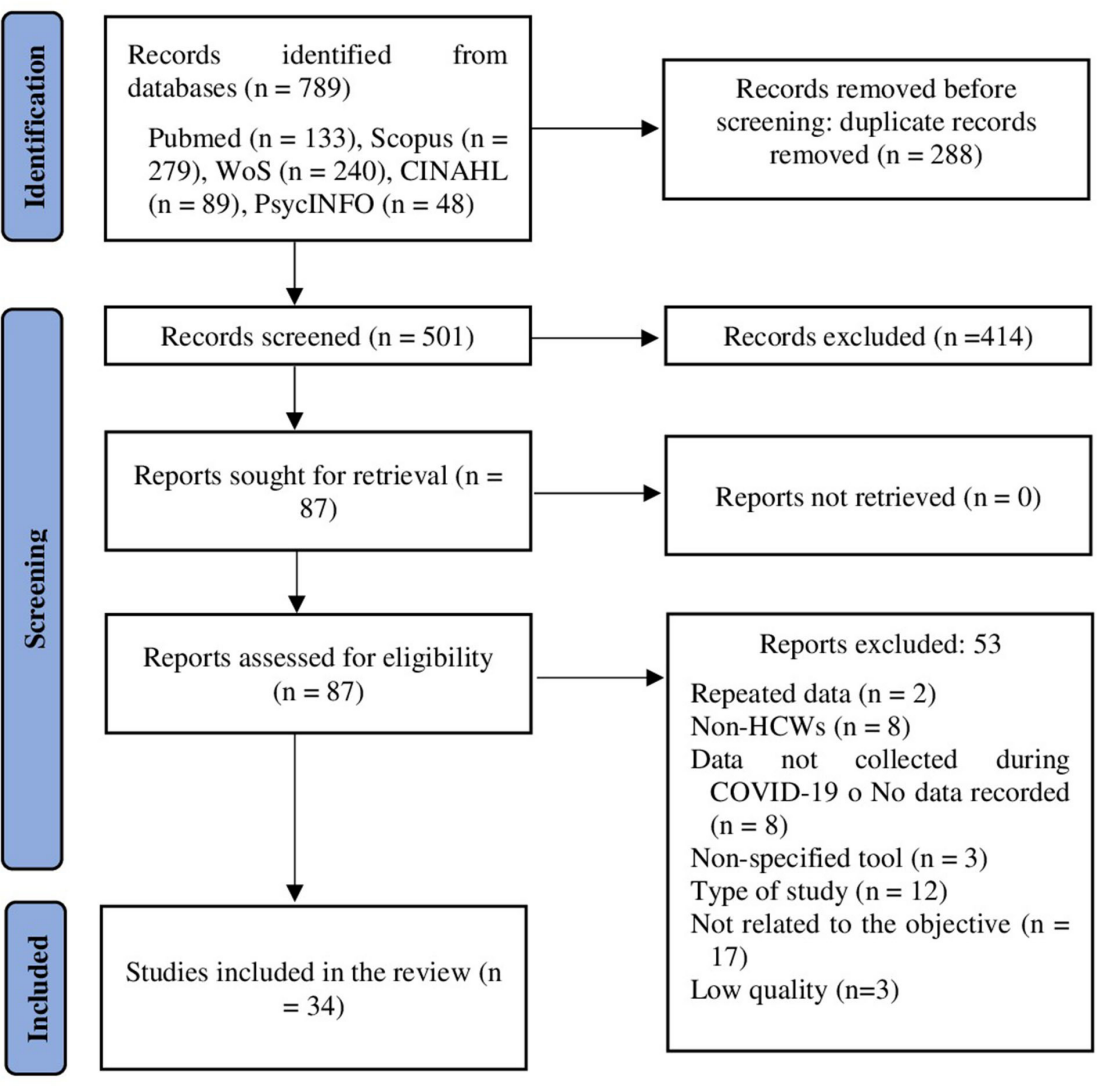
Identification of studies *via* databases (PRISMA flow diagram).

The primary search in the databases yielded 789 citations. After eliminating duplicates (*n* = 288), titles and abstracts of 501 articles were screened. Of these, 414 citations were discarded upon reviewing title and abstract. After having read the full text of the 87 remaining citations, 53 were discarded, either because they contained repeated data (*n* = 2), the population was not HCWs (*n* = 8), the data were collected before the pandemic by COVID-19 or the date of collection was not stated (*n* = 8), the tool was not detailed (*n* = 3), because of the type of study (*n* = 12), because the study was not related to the objective (*n* = 17), and for low quality (*n* = 3). The flow diagram is presented in Figure 1.

### Study characteristics

Table 4 presents the study characteristics of the 34 selected articles. Of the total number of studies, five were conducted in China, five in Spain, four in the United States, four in the United Kingdom, two in Australia, and one in other countries (Bangladesh, Belgium, Brazil, Chile, Colombia, Egypt, Germany, India, Iran, Malaysia, Pakistan, Republic of Ireland, Russia, and Turkey). Regarding the data collection period, in 32 of the 34 studies it was carried out during the year 2020, preferably in the first and second quarter of the year, coinciding with the first wave of the pandemic. Regarding the sample, 5 of the 34 studies only included nurses and 3 of the 34, only physicians; the rest included two or more healthcare professions. Finally, regarding the measurement instrument, 12 of the 34 studies used item 9 of the PHQ-9, 8 of the 34 used the C-SSRS, 5 used a dichotomous question on whether they had suicidal thoughts, and the rest used other instruments (SSEV, question 17 of the SRQ-20, BSSI, CES-D-SI, SIS, CIS-R, Self-Injurious Thoughts and Behavior Interview, and SSI).

**Table 4:** Characteristics of the studies included in the systematic review.

**Studies**	**Context**	**Main aim**	**Type of study**	**Population**	**Instrument**	**Main outcomes**	**JBI**
Brady et al. (31)	Republic of Ireland (November 2020 to January 2021)	To quantify the mental health of nursing home staff during the COVID-19 pandemic in the Republic of Ireland	Cross-sectional study	390 nurses	C-SSRS	Suicidal ideation and suicide planning were reported, respectively, by 13.8% (95% CI, 10.4−17.3%) and 9.2% (95% CI, 6.4−12.1%) of participants with no between group differences.	7/8
Bismark et al. (32)	Australia (August–October, 2020)	To identify the prevalence and predictors of (a) thoughts of suicide or self-harm among HCWs during the COVID-19 pandemic and (b) help-seeking among those HCWs with thoughts of suicide or self-harm.	Cross-sectional study	7,795 HCWs	PHQ-9 (question 9)	10.5% of HCWs reported thoughts of suicide or self-harm. HCWs with these thoughts experienced higher rates of depression, anxiety, post-traumatic stress disorder and burnout than their peers. The odds of suicide or self-harm thoughts were higher among HCWs who had friends or family infected with COVID-19 (O*R =* 1.24, 95% CI = [1.06, 1.47]), were living alone (O*R =* 1.32, 95% CI = [1.06, 1.64]), younger (?30 years cf. >50 years; O*R =* 1.70, 95% CI = 1.36-2.13), male (O*R =* 1.81, 95% CI = [1.49, 2.20]), had increased alcohol use (O*R =* 1.58, 95% CI = [1.35, 1.86]), poor physical health (O*R =* 1.62, 95% CI = [1.36, 1.92]), increased income worries (O*R =* 1.81, 95% CI = [1.54, 2.12]) or prior mental illness (O*R =* 3.27, 95% CI = [2.80, 3.82]). Having dependent children was protective (O*R =* 0.75, 95% CI = [0.61, 0.92]). Fewer than half (388/819) of the HCWs who reported thoughts of suicide or self-harm sought professional support.	7/8
Höller et al. (33)	Germany (February–April, 2021)	To examine (1) the psychological burden and (2) suicidal ideation and its associated risk factors one year after the COVID- 19 pandemic begun	Cross-sectional study	1,311 nurses	SSEV	21.7% of HCWs reported recent (in the last 4 weeks) suicidal ideation and 0.5% reported a recent suicide attempt. 44.5% of HCWs reported lifetime suicidal ideation and 12.6% reported at least one life-time suicide attempt. Only depression, perceived burdensomeness, agitation and previous lifetime suicide attempt were associated with suicidal ideation. No differences in suicidal ideation were found between nurses with versus without direct contact with people with COVID-19.	7/8
Oliveira et al. (34)	Brazil (June–July, 2020)	To identify the prevalence of and factors associated with suicidal ideation among nursing professionals from a municipality in southern Brazil.	Cross-sectional study	890 nurses	SRQ-20 (question 17)	The observed prevalence of suicidal ideation was 7.4%. Suicidal ideation was inversely related to per capita income > 3 minimum monthly wages (PR: 0.28; 95% CI: 0.11–0.68), and positively related to the use of psychotropic drugs (PR: 3.14; 95% CI: 1.87–5.26). In addition, suicidal ideation was also associated with assessing one's working conditions as poor (PR: 2.16; 96% CI: 1.13–4.13), reporting a heavy burden at work (PR: 1.93; 95% CI: 1.08–3.43), reporting increased burden post-pandemic (PR: 2.03; 95% CI: 1.13–3.64), and problems with alcohol (PR: 2.56; 95% CI: 1.31–4.96).	8/8
Mortier et al. (24)	Spain (May–September, 2020 and October–December, 2020)	To estimate four-month STB incidence among Spanish HCW active during the first wave of the Spain COVID-19 pandemic; and to investigate individual-and population-level associations of a wide range of potential risk factors with STB incidence.	Cross-sectional study	4,809 HCWS	C-SSRS (modified)	Suicidal thoughts and behaviors incidence was estimated at 4.2%. Risk factors significantly associated with suicidal thoughts and behaviors incidence were pre-pandemic lifetime mental disorders (OR range 1.59–2.53), being an auxiliary nurse (O*R =* 2.07), being single, divorced, legally separated, or widowed (O*R =* 1.72). Having a pre-pandemic monthly income level higher than 2200€ was a protective factor (OR range 0.49–0.53). Interpersonal stress (OR range = 1.23–1.57) was strongly associated with STB, followed by personal health-related stress and stress related to the health of loved ones (OR range 1.30–1.32), and by the perceived lack of preparedness of the healthcare center (O*R =* 1.34). Other significantly associated risk factors were financial factors (OR range 1.26–1.81), having been isolated or quarantined for COVID-19 (O*R =* 1.53), and having changed to a specific COVID-19 related work location (O*R =* 1.72).	7/8
Ortiz-Calvo et al. (35)	Spain (April–June, 2020)	To study the potential effect of self-perceived social support and resilience on the mental health outcomes of a large sample of HCWs from Spain during the initial COVID-19 pandemic outbreak	Cross-sectional study	2,372 HCWS	C-SSRS (modified)	The rate of death thoughts was 7%, and higher among HCWs who reported a history of prior mental health problems (21%). Resilience and self-perceived social support were inversely associated with death thoughts. Death thoughts did not show a clear age pattern. Women reported death thoughts more frequently than men. Frontline HCWs reported distinctly higher presence of death thoughts.	7/8
Que et al. (36)	China (May–July, 2020)	To examine the relationship between COVID-19-related traumatic event exposure and suicidal ideation among hospital HCWs, and identify mediating roles of sleep disturbances in this relationship	Cross-sectional study	16,220 hospital HCWs	PHQ-9 (question 9)	13.3% of HCWs reported suicidal ideation n the past month. Insomnia severity (β = 0.309, *p < * 0.001), nightmare frequency (β = 0.455, *p < * 0.001), depressive symptoms (β = 0.358, *p < * 0.001), and anxiety symptoms (β = 0.371, *p < * 0.001) were positively associated with the risk of suicidal ideation.	7/8
Salman et al. (37)	Pakistan (first wave)	To assess suicidal ideation and its predictors among Pakistani HCWs during the early phase of the COVID-19 pandemic	Cross-sectional study	398 HCWs	PHQ-9 (question 9)	14.3% prevalence of suicidal ideation among Pakistani HCWs. Participants' occupation, their duty during the pandemic, working experience, anxiety and depression were found be associated with suicidal ideation (*p < * 0.05). HCWs directly engaged in managing COVID-19 patients were 2.25 times more likely to have suicidal ideation than the second-line health professionals.	7/8
Abdelghani et al. (38)	Egypt (March–May, 2020)	To explore the newly termed phenomenon, coronaphobia, and identify its associated correlates among physicians during their battle against the COVID-19 pandemic in Egypt.	Cross-sectional study	426 physicians	A question was asked (Yes/No)	10.1% of HCWs reported self-harm thoughts during pandemic. Frontline workers had more self-harm thoughts, compared to second-line workers (13.6% vs. 9.4%, respectively). Excessive anxiety and fears of COVID-19 virus infection were found to be associated with suicidal thoughts and intense feelings of hopelessness	6/8
Al-Humadi et al. (39)	United States (April–May, 2020)	To establish the incidence of depression, suicidal thoughts, and burnout; to identify factors associated with the development of these mental health issues; to examine differences between attending and resident physicians; and to examine differences between female and male physicians	Cross-sectional study	225 physicians	PHQ-9 (question 9)	The rate of suicidal ideation for attending and resident/fellow physicians was 7.1% and 6.2%, respectively. No difference was found between resident/fellow and attending physician rates of suicidal ideation (*t =* 0.641; *P =* 0.522). 5.8% of married participants presented suicidal ideation, compared to 7.4% of single workers. Rate of suicidal ideation for female resident/fellow and attending physicians was 6.9% and 5.6%, respectively. Internal medicine and other non-surgical specialties had the highest rates of suicidal ideation (10.2%). Suicidal ideation was positively associated with number of times on call in the last month (OR: 1.17, 95% CI [1.04, 1.32], *P =* 0.02), a history of being diagnosed or treated for depression or anxiety (OR: 1.17, 95% CI [1.04, 1.32], *P =* 0.01), and younger age (OR: 0.07, 95% CI [.04, 0.14], *P =* 0.05).	8/8
Alvarado et al. (40)	Chile (May–August, 2020)	To evaluate HCWs' mental health and its associated factors during the pandemic in Chile	Cross-sectional study	1,934 HCWs	C-SSRS	12.2% of respondents reported wishing that they were dead. Among women, this figure was significantly higher (13.6%) than among men (8.0%). Suicidal ideation was reported by 2.3% of participants, with no significant differences by sex or place of work. HCWs who reported a wish to be dead scored significantly higher on the GHQ-12 (22.6 ± 5.8 vs. 15.4 ± 5.9; *p < * 0.001) and the PHQ-9 (15.1 ± 5.9 vs. 7.7 ± 5.0; *p < * 0.001).	8/8
Amsalem et al. (41)	United States (September–December, 2020)	To examine rates of depression, GAD, PTSD, and moral injury among United States HCWs in the COVID-19 era.	Longitudinal study (0, 30, and 90 days)	350 HCWs	PHQ-9 (question 9)	65/350 participants (19%) reported suicidal thoughts and 35 of them (10% of the entire sample) endorsed “several days”. Rates of PHQ-9 depression and suicidal ideation did not significantly change over time (*F =* 2.4, *P =* 0.091, and *F =* 1.9, *P =* 0.149, respectively).	7/8
Ariapooran et al. (19)	Iran (2020)	To evaluate the prevalence of Secondary Traumatic Stress and comparing depression, anxiety, and suicidal ideation in nurses with and without STS symptoms during the COVID-19 outbreak	Cross-sectional study	315 nurses (hospitals)	BSSI	There were inter-group differences in nurses with and without STS symptoms regarding Suicidal Ideation (*F =* 2.424; *p < * 0.091). Nurses with STS symptoms received higher scores in depression, anxiety, and SI than the ones without STS symptoms.	6/8
Bruffaerts et al. (42)	Belgium (April–July, 2020)	To examine the prevalence of STB in HCWs in Belgium, the country with the highest suicide rate within Europe	Cross-sectional study	6,409 HCWs	C-SSRS (modified version)	Prevalence was 3.6% death wish, 1.5% suicide ideation, 1.0% suicide plan, and 0.0% suicide attempt (*n =* 2). Also, substance use disorder or post-traumatic stress disorder (PTSD) were more than twofold associated with suicide ideation and/or plan.	7/8
Campo-Arias et al. (43)	Colombia (October—November, 2020)	To examine the association of perceived discrimination related to COVID-19 with psychological distress in HCWs in the Colombian Caribbean region	Cross-sectional study	150 HCWs	CES-D-SI	Perceived discrimination scores showed positive correlations with suicide risk in nursing assistants (r_s_ = 0.35) and physicians (r_s_ = 0.31).	6/8
Dobson et al. (44)	Australia (April–May, 2020)	To examine psychological distress in healthcare workers (HCWs) during the COVID-19 pandemic in April-May 2020.	Cross-sectional study	320 HCWs	PHQ-9 (question 9)	Twenty-three participants (8.1 %) reported suicidal ideation during the 2-week reporting period, being higher among nurses (14.7%), among men (21.7%), and among HCWs not on the frontline (7.6%).	7/8
Duru (45)	Turkey (January 2019–January 2020 and January–April, 2021)	To evaluate the effect of the COVID-19 pandemic on the physical well-being and mental health of ICU HCWs	Cross-sectional study	51 ICU HCWs tertiary care hospital	SIS	SIS scores indicate absence of suicidal ideation. A change in Vit D levels was positively correlated with SIS scores (*r =* 0.381, *P =* 0.006).	8/8
Greenberg et al. (46)	UK (June–July, 2020)	To identify the rates of probable mental health disorder in staff working in ICUs in nine English hospitals during June and July 2020.	Cross-sectional study	709 ICU HCWs	PHQ-9 (question 9)	13% of respondents reported having thoughts that [they] would be better off dead, or of hurting [themselves] in some way, several days or more frequently in the past 2 weeks. A significantly higher proportion of nurses (19%) than physicians (8%) or other clinical staff (10%) (χ^2^ = 26.8, degrees of freedom [df] = 8, *P < * 0.002) reported these thoughts.	8/8
Hong et al. (47)	China (February, 2020)	To assess the immediate psychological impact on frontline nurses in China.	Cross-sectional study	4,692 Frontline nurses	PHQ-9 (question 9)	About 6.5% respondents had suicidal ideation. A poorer subjective health (poor: O*R =* 7.56; fair: O*R =* 3.38), not enough support from family (O*R =* 2.05) or hospital authorities (O*R =* 1.54), and less opportunities for reflecting opinions through mass media (O*R =* 1.47) were shown to be risk factors. Family member not infected (O*R =* 0.15) and lower job-related stress (low: O*R =* 0.40; medium: O*R =* 0.61) had protective effects on suicidal ideation	8/8
Lamb et al. (48)	UK (April–June, 2020)	To report preliminary findings on the prevalence of, and factors associated with, mental health and well-being outcomes of HCWs during the early months (April–June) of the COVID-19 pandemic in the UK	Cross-sectional study	4,378 HCWs (Non-clinical 32.3%)	CIS-R	In the past 2 months, 8.5% (95% CI 7.3 to 9.8) of participants had considered taking their own life, while 2.0% (95% CI 1.4 to 2.7) had attempted suicide, and 3.0% (95% CI 2.3 to 3.9) had harmed themselves.	8/8
Majumder et al. (49)	UK (April–May, 2020)	To explore the effects of the pandemic on the psychological wellbeing of UK HCWs, as well as the coping mechanisms used and the workplace support that they found helpful	Cross-sectional study	533 HCWs	*Ad hoc* questionnaire	2.6% reported thoughts of self-harm (2.3% frontline vs. 3.9 non-frontline) and 1.7% experienced suicidal thoughts (1.6% frontline vs. 1.9 non-frontline). No statistically significant differences were found in any of the cases.	6/8
Mediavilla et al. (50)	Spain (May–June, 2020)	To explore the association between perceived discrimination and mental health outcomes in a large sample of HCWs in Spain	Cross-sectional study	2,053 HCWs	C-SSRS	5.6% reported death thoughts. Perceived discrimination was associated with a 2-fold increase in risk of reporting death thoughts (O*R =* 2.0, 95 percent CI: 1.4, 3.1).	7/8
Mediavilla et al. (51)	Spain (Aprol–June, 2020)	To analyse the association between three work-related stressors and mental health outcomes in a large sample of Spanish HCWs during the initial COVID-19 outbreak.	Cross-sectional study	2,370 HCWs	C-SSRS	7% reported death wishes and 17% of them reported active suicidal ideation. Death wishes were also more frequent among those who changed their job functions. Prior history of mental health problems was associated in adjusted models with the probability of reporting death wishes (O*R =* 3.9, 95% CI: 2.3, 6.3).	7/8
Mortier et al. (52)	Spain (Mar–Jul, 2020)	To investigate the prevalence and correlates of suicidal thoughts and behaviors among hospital HCWs during the first wave of the Spain COVID-19 outbreak	Cross-sectional study	5,450 HCWs	C-SSRS (modified)	Thirty-day suicidal thoughts and behaviors prevalence was estimated at 8.4% (4.9% passive ideation only, 3.5% active ideation with or without a plan or attempt). A total of *n =* 6 professionals attempted suicide in the past 30 days. In adjusted models, 30-day suicidal thoughts and behaviors remained significantly associated with pre-pandemic lifetime mood (O*R =* 2.92) and anxiety disorder (O*R =* 1.90). Significant modifiable factors included a perceived lack of coordination, communication, personnel, or supervision at work, and financial stress.	7/8
Mosolova et al. (53)	Russia (May, 2020 and Oct, 2020)	To assess the range of psychopathological symptoms and risk factors in frontline HCWs during spring and autumn outbreaks of the new coronavirus infection in Russian Federation	2 Cross-sectional study	2,195 HCWs	PHQ-9 (question 9)	2.4% of HCWs reported suicidal thoughts. Risk factors female gender, younger age, being a physician, working for over a week, living outside of Moscow or Saint Petersburg, being vaccinated against COVID-19.	7/8
Murata et al. (54)	United States (April–July, 2020)	To assess its mental health impact across the lifespan in the United States in adolescents, adults, and HCWs	Cross-sectional study	1,672 HCW	Self-Injurious Thoughts and Behavior Interview	In HCWs the prevalence was 5.6% lifetime non-suicidal self-injurious behavior, 4.0% lifetime suicidal ideation, 18% lifetime actual suicide attempt and 19% lifetime suicidal ideation or behavior.	7/8
Parthasarathy et al. (55)	India (July–September, 2020)	To examine whether the nature of occupation, socio-demographic variables, life-style, family support, substance use and suicidality correlate with anxiety and depression among HCWs	Cross-sectional study	5,995 HCWs	2 questions: Suicidal thoughts and attempts (Yes/No)	HCWs with anxiety and depression have reported an increase in suicidal thoughts but not attempts after the onset of the pandemic. HCWs with anxiety and depression have reported an increase in suicidal thoughts but not attempts after the onset of the pandemic.	7/8
Sahimi et al. (27)	Malaysia (March 2020)	To investigate suicidal ideation in terms of the rate and associated factors in a sample of Malaysian HCWs during the early-phase of the COVID-19 pandemic.	Cross-sectional study	171 HCWs	PHQ-9 (question 9)	The proportion of HCWs with current suicidal ideation was 11.1%. Factors significantly associated with current suicidal ideation were single status (*P =* 0.017), higher levels of health anxiety (*P =* 0.234), and higher severity of depression (*p < * 0.001). Participants with more than 10 years of service duration had a significantly lower rate of current suicidal ideation (*P =* 0.013). Clinical depression was the most significant factor associated with current suicidal ideation (*p < * 0.001, O*R =* 55.983, 95% CI = 9.015–347.671) followed by mild (subthreshold) depression (*P =* 0.001, O*R =* 115.984, 95% CI = 2.977–85.804). Service duration of more than 10 years was associated with significantly less suicidal ideation (*P =* 0.049, O*R =* 0.072, 95% CI = 0.005–0.993).	8/8
Xu et al. (56)	China (February–March, 2020)	To investigate the prevalence of suicidal and SSI and its related factors in hospital staff during the COVID-19 pandemic.	Cross-sectional study	11,507 HCWs 46 hospitals	A question was asked about SSI and PHQ-9 (question 9)	6.47% (744) of the hospital staff reported SSI. The SSI prevalence in doctors, nurses, technicians, and administrators were 6.26%, 6.68%, 6.37%, and 5.56%, respectively. Marital status, work hours per day, sleep hours per day, frontline department family members or relatives infected, community members infected, probability of infection, willingness to work in a COVID-19 ward, attendance of parties, concerns on COVID-19 progress, confidence in defeating COVID-19, prediction for lasting time, almost all the psychological characteristics and most items in perceived stress and support scales showed significant differences between hospital staff with and without SSI (P < 0.05).	7/8
Young et al. (18)	United States (April, 2020)	To quantify the rates of psychological distress among HCWs during the COVID-19 pandemic and to identify job-related and personal risk and protective factors	Cross-sectional study	1,326 HCWs	PHQ-9 (question 9)	5% (64 of 1,326) endorsed suicidal ideation. Those respondents with a self-reported psychiatric history reported more frequent suicidal ideation than those without such history (48 of 572 [8%] vs. 16 of 754 [2%], respectively; p < 0.001).	8/8
Cai et al. (57)	China (February, 2020)	To compare the psychological impact of the COVID-19 outbreak between frontline and non-frontline medical workers in China	Case-control	1,173 frontline and 1,173 non-frontline medical workers	A question was asked (“Once/several times” or “Never”)	No significant difference was observed in terms of suicidal ideation (12.0% vs. 9.0%, adjusted O*R =* 1.25, 95% CI = 0.92–1.71) between frontline medical workers than non-frontline medical workers.	10/10
Mamun et al. (58)	Bangladesh (April, 2020)	To investigate the suicidality and its associated risk factors of HCWs by comparing with that of general population as it is anticipated that the HCWs may have higher suicidality as of being exposed to critical situation and higher mental health sufferings	Cross-sectional study	834 HCWs	A question was asked (Yes/No)	About 6.0% of HCWs had suicidal behavior, with no detectable differences within the groups (i.e., general population and HCWs). Regression analysis showed that being female, being divorced, and having no child were emerged as independent predictors for suicidality. There was no significant association between the personal protective equipment related or patient-care related variables and suicidal behavior of the HCWs. Majority of the participants sometimes had fear of death although no significant relation of the factor was found with suicidality.	6/8
Rathod et al. (59)	UK (May–July, 2020 and Oct-Dec, 2020)	To investigate the psychological impact of COVID-19, resultant restrictions, impact on behaviors and mental wellbeing globally	Cross-sectional study	3,933 HCWs	A question was asked (Yes/No)	Most of the key HCWs have higher likelihood of suicidal thoughts and worries about coronavirus compared to others. Suicidal thoughts increase amongst almost all individuals with pre-existing health conditions. Individuals with pre-COVID-19 suicidal thoughts show lower likelihood of following government advice, communications with friends and family, coping activities, confidence on coping, but higher likelihood of doing risky activities, with higher scores on PHQ-9, GAD-7, and IES-R.	7/8
Xiaoming et al. (60)	China (February, 2020)	To investigate the psychological status of hospital HCWs and provide references for psychological crisis intervention in the future	Cross-sectional study	8,817 hospital HCWs	SSI	The prevalence of SSI was 6.5%. Various epidemic-related attitudes and behaviors were independent factors for SSI, such as the need for psychological assistance before or during the epidemic (O*R =* 1.826, 95% CI = 1.310–2.545; O*R =* 2.277, 95% CI = 1.636–3.171), unconfident about defeating COVID-19 (O*R =* 2.435, 95% CI = 1.184–5.005), ignorance about the epidemic (O*R =* 2.559, 95% CI = 1.451–4.531), willingness of attending parties (O*R =* 2.235, 95% CI = 1.339–3.731), and poor self-rated health condition (O*R =* 5.228, 95% CI = 3.650–7.489) among hospital HCWs (P < 0.05).	8/8

JBI, Joanna Briggs Institute; HCWs, Healthcare Workers; OR, Odds Ratio; CI, Confidence Interval; UK, United Kingdom; GAD, Generalized Anxiety Disorder; PTSD, Posttraumatic Stress Disorder; PHQ-9, Patient Health Questionnaire (9 items); BSSI, Beck Scale for Suicidal Ideation; C-SSRS, Columbia Suicide Severity Rating Scale; CES-D-SI, Suicidal Ideation Scale of the Center for the Epidemiological Study of Depression; SIS, Suicidal Ideation Scale; SSEV, Suicide Ideation and Behavior Scale; SSI, Self-Harm Ideation; CIS-R, Clinical Interview Schedule; SRQ-20, Self-Reporting Questionnaire.

The included studies were assessed with the JBI critical appraisal tool, where high mean scores were obtained in all the finally included studies.

### Main findings

Suicidal thoughts were reported by 21.7 to 2.4% of HCWs. 0.5 to 12.6% reported at least one lifetime suicide attempt, 0.5 to 3.5% reported a recent suicide attempt, and 3.0 to 0.5% had self-harmed (33, 48, 54).

Major factors associated with increased suicidality include higher rates of depression (27, 32, 33, 36, 37, 39, 40, 55); anxiety (19, 27, 32, 36–39, 52, 55); post-traumatic stress disorder (32, 42); pre-pandemic lifetime mental disorders (24) or previous lifetime suicide attempt; insomnia severity; nightmare frequency; poorer subjective health (32, 33, 36, 47, 60); and burnout (32). In addition, other personal factors predisposing to suicidal thoughts include being female (58); having friends or family members infected with COVID-19 (24, 32, 56); living alone; having poor physical health (32); being single (27, 39), divorced, legally separated, or widowed (24); higher alcohol consumption (32, 34); psychotropic drug use (34, 42); and change in vitamin D levels (45). Younger age is considered a risk factor for some participants (32, 35), a protective factor for others (39), and shows no clear age pattern for the rest (35). Regarding work-related factors during the pandemic, suicidal ideation was associated with financial concerns (24, 32, 34), assessing one's working conditions as poor (34) or perceived lack of preparedness of the health care facility (24); having had job functions changed (51); having been isolated or quarantined for COVID-19 (24, 56); having been moved to a specific workplace related to COVID-19; being an auxiliary nurse (24) or a nurse (44, 46); reporting high workload (34) or stress (56); feeling perceived discrimination (43, 50) and not feeling support from family (47) or superiors (52); and reporting an increased post-pandemic burden (34). As for HCWs coping with COVID-19, no differences in suicidal ideation were found between nurses with and without direct contact with persons with COVID-19 (33, 49, 57), although another study presents discrepancies indicating that frontline HCWs reported a clearly higher presence of thoughts of death (35, 37, 38).

Finally, factors such as having dependent children (32), having a per capita income of more than three minimum monthly wages (34) or higher than 2200 euros (24), resilience and self-perceived social support (35), not having an infected family member, and lower work stress had protective effects on suicidal ideation (47). In addition, HCWs with more than 10 years of service had a significantly lower rate of current suicidal ideation (27).

## Discussion

This study sought to review the factors that may protect or predispose HCWs to suicide attempts and suicidal ideation during the COVID-19 pandemic. The results suggest that there are a number of personal, social, and occupational factors that may predispose HCWs to develop and tend to suicidal ideation, as well as others that may reduce the number of suicidal thoughts and tendencies, such as support systems and certain personal factors.

In previous studies, suicidal behaviors were found to have increased during the pandemic in the general population and in samples of HCWs (61). This already corroborates findings from pre-pandemic studies where suicide rates among HCWs were already higher than those reported in the general population, with differences between men and women, especially among female physicians. There is a wide variability in the prevalence of suicidal ideation and suicide attempts in the different samples consulted within a COVID-19 (38) pandemic setting. This phenomenon could be explained by the variability of the samples, by the impact of disease control measures in each country, as well as by the difference in the levels of stress, anxiety, fear, and depression experienced by HCWs during the pandemic (62). Indeed, in the meta-analytic study by Dragioti et al. (63) of one hundred and seventy-three studies conducted between February and July 2020, the COVID-19 pandemic was found to have a greater impact on mental health in people living in low-income countries, in those who had adopted more restrictive measures, and in more vulnerable populations. Rudenstine et al. (64) consider that the risk factors influencing the general population are those that affect material and economic variables, the social level, and those related to accessing vital resources. In the case of HCWs, these factors may have a different relevance during the pandemic and others may become more relevant, so synergistic relationships may be established between them (55).

In this line, there are a number of factors associated with higher suicidality such as higher rates of depression (27, 32, 33, 36, 37, 39, 40, 55); anxiety (19, 27, 32, 36–39, 52, 55); post-traumatic stress disorder (32, 42); pre-pandemic lifetime mental disorders (24) or previous lifetime suicide attempt; insomnia severity; nightmare frequency; a poorer self-perceived health (32, 33, 36, 47, 60); and burnout (32). In most cases, all these risk factors have increased in impact during the COVID-19 pandemic and, as Mamun and Ullah (65) estimate, approximately 90% of suicides are due to psychological distress in the face of continued exposure to highly stressful situations.

Although most suicidal ideation is due to problems related to psychological distress, the explanation may be varied and multicausal (60, 66). HCWs have been repeatedly exposed to death and pain during the COVID-19 pandemic (67), and as postulated by Smith and Cukrowicz (68), constant exposure to pain and death may favor suicidal behavior and ideation. This may suggest that work environments where there is a higher risk of infection may favor a worsening of the mental health of particularly exposed HCWs (69). In this case, there is certain controversy as to the higher or lower prevalence of suicide rates among frontline HCWs compared to other types of workers. Some studies (33, 49, 57) have found no statistically significant differences in terms of suicidal ideation rates between HCWs working on the front line and those without direct contact. Others, on the other hand, have indeed found differences in this regard (24, 35, 37, 38). In the latter case, this could be justified by a change in the work environment, the functions to be carried out, and a greater perception of risk in relation to the disease. Studies such as the one by Salman et al. (37) estimated that HCWs working on the front line are up to 2.25 times more likely to have suicidal ideation. Changing functions or work location has also been considered by Mediavilla et al. (51) as another risk factor, since it can worsen the mental health of HCWs. In fact, even before the pandemic, there were services in which suicide rates were higher than in others, such as the case of HCWs working in the operating room (70). Other authors found that Internal medicine and other non-surgical specialties had the highest rates of suicidal ideation (39). Other factors such as having been hospitalized due to COVID-19 infection, having had family members infected with COVID-19, and self-perceived probability of contracting COVID-19 may be predisposing factors to the uncertainty caused by the disease (71). In this regard, in a case study of press reports, being infected with COVID-19 was the most common reported reason for suicide, followed by work-related stress, fear of COVID-19 infection, fear of transmitting the virus to others, anxiety about witnessing overwhelming death, and mental distress (14).

On the other hand, a series of factors do not show a clear trend between studies. This is the case of age, sex, or the type of HCW studied. As indicated by Mamun et al. (58), Alvarado et al. (40), Jahan et al. (14), and Mosolova et al. (53), being female may be a risk factor that increases the rates of suicidal ideation compared to males, but other studies such as the one by Bismark et al. (32) and another one by Dobson et al. (44) differ from these conclusions and postulate that males offer higher ideation rates than females. To overcome this dichotomy, homogeneous samples should be compared to avoid possible biases. Being younger is considered a risk factor for some studies (32), a protective factor for others (39), and shows no clear age pattern for the rest (35). In relation to the type of role of the HCW, the prevalence of suicidality may vary. In the study by Mortier et al. (52) in Spain, an auxiliary nurse was 2 times more likely to develop suicidal thoughts and behaviors. In the case of the study by Greenberg et al. (46), 1 in 5 nurses reported suicidal thoughts compared to 1 in 10 physicians. Likewise, in the study by Mosolova et al. (53), the group with the highest risk was that of physicians, compared to the rest of HCWs. In contrast, in the study by Xu et al. (56), physicians, nurses, technicians, and administrators showed a similar prevalence. In this vein, the study by Dobson et al. (44) carried out in Australia (April–May, 2020) on a sample of 320 HCWs, suicidal ideation was more present among nurses. The differences between groups may respond to the fact that nurses may have a more intense nurse-patient relationship than other professionals because of the long hours they spend with patients.

Just as some factors have proven to be predisposing to suicidal tendencies and behaviors, there are others that can be considered protective. In many cases, these factors are related to the support system that the HCW has at the individual, family, and work level. Factors such as having dependent children (32), per capita income > 3 minimum monthly wages (34) or higher than 2200 euros (24), resilience and self-perceived social support (35), no family member infected, and lower job-related stress had protective effects on suicidal ideation (47). In addition, HCWs with more than 10 years of service duration had a significantly lower rate of current suicidal ideation (27).

All in all, the findings of this review show that the context surrounding the COVID-19 pandemic at the social, occupational, family, personal, and public health levels may have had an impact on suicidal ideation and suicide attempts in the general population and, in particular, in healthcare workers as a result of the factors they are exposed to in their professional performance and the social and healthcare context that surrounds them. It is true, though, as previously mentioned, that suicidal ideation and suicide attempts will not only depend on the extrinsic factors that affect the healthcare worker in a pandemic context, but internal factors related to previous health problems, adequate support networks, financial solvency, among others, are also particularly relevant.

### Limitations

This systematic review is not without limitations. Firstly, most of the included studies were cross-sectional and used hetero-administered instruments *via* online surveys. In this sense, population characteristics, methodological differences, heterogeneous samples, etc. meant that the resulting findings were very heterogeneous. Thirdly, the timing of data collection and measures of confinement/isolation was different in each study, hence the data were considered inadequate for meta-analysis. Finally, the health system, the allocation of resources for mental health promotion, as well as the adoption of preventive measures adopted by different countries may differ, and these deviations may influence the comparability of indicators.

## Conclusion

There are a number of underlying factors such as higher rates of depression, anxiety, pre-pandemic lifetime mental disorders or previous lifetime suicide attempt, living alone, having problems with alcohol and/or other drugs, etc. that favor the emergence of suicidal tendencies and ideation in times of COVID-19. Similarly, there are a series of factors that the pandemic may have precipitated, such as economic concerns, assessing one's working conditions as poor, having infected family members or friends, changes in services or functions, feeling discriminated against or stigmatized by society. Other factors such as age, sex, or type of HCW differ between studies.

## Data availability statement

The raw data supporting the conclusions of this article will be made available by the authors, without undue reservation.

## Author contributions

FF-C, RA-C, JG-S, and JV-L: conceptualization and writing–review and editing. BP-C, JV-L, and JG-I: data curation. BP-C, FF-C, RA-C, JV-L, and JG-I: formal analysis. BP-C and FF-C: investigation. RA-C, JG-S, JV-L, and JG-I: methodology. RA-C, JG-S, and JG-I: project administration. BP-C, FF-C, RA-C, JG-S, JV-L, and JG-I: resources. BP-C and RA-C: software. RA-C, JG-S, and JV-L: supervision. FF-C, RA-C, JG-S, and JG-I: validation. BP-C, JG-S, JV-L, and JG-I: visualization. BP-C, FF-C, and JG-I: writing–original draft. All authors contributed to the article and approved the submitted version.

## Funding

Funding for open access charge: Universidad de Huelva/CBUA.

## Conflict of interest

The authors declare that the research was conducted in the absence of any commercial or financial relationships that could be construed as a potential conflict of interest.

## Publisher's note

All claims expressed in this article are solely those of the authors and do not necessarily represent those of their affiliated organizations, or those of the publisher, the editors and the reviewers. Any product that may be evaluated in this article, or claim that may be made by its manufacturer, is not guaranteed or endorsed by the publisher.
