# Supplementary material for: Suicidal ideation and suicide attempts in healthcare professionals during the COVID-19 pandemic: A systematic review
Source: Front Public Health. 2022 Dec 6;10:1043216. doi: 10.3389/fpubh.2022.1043216 (PMC9767440; doi:10.3389/fpubh.2022.1043216)
Supplement: Supplementary file 1 [file Data_Sheet_1.doc]

**Table S1.** Overall appraisal of Cross-Sectional Studies.

| **Study** | **JBI** | **Were the criteria for inclusion in the sample clearly defined?** | **Were the study subjects and the setting described in detail?** | **Was the exposure measured in a valid and reliable way?** | **Were objective, standard criteria used for measurement of the condition?** | **Were confounding factors identified?** | **Were strategies to deal with confounding factors stated?** | **Were the outcomes measured in a valid and reliable way?** | **Was appropriate statistical analysis used?** |
| --- | --- | --- | --- | --- | --- | --- | --- | --- | --- |
| Brady et al., 2022 | 7/8 |  |  |  |  |  |  |  |  |
| Bismark et al., 2022 | 7/8 |  |  |  |  |  |  |  |  |
| Höller et al., 2022 | 7/8 |  |  |  |  |  |  |  |  |
| Kantorski et al., 2022 | 8/8 |  |  |  |  |  |  |  |  |
| Mortier et al., 2022 | 7/8 |  |  |  |  |  |  |  |  |
| Ortiz-Calvo et al., 2022 | 7/8 |  |  |  |  |  |  |  |  |
| Que et al., 2022 | 7/8 |  |  |  |  |  |  |  |  |
| Salman et al., 2022 | 7/8 |  |  |  |  |  |  |  |  |
| Abdelghani et al., 2021 | 6/8 |  |  |  |  |  |  |  |  |
| Al-Humadi, et al., 2021 | 8/8 |  |  |  |  |  |  |  |  |
| Alvarado et al., 2021 | 8/8 |  |  |  |  |  |  |  |  |
| Amsalem et al., 2021 | 7/8 |  |  |  |  |  |  |  |  |
| Ariapooran et al., 2021 | 6/8 |  |  |  |  |  |  |  |  |
| Bruffaerts et al., 2021 | 7/8 |  |  |  |  |  |  |  |  |
| Campo-Arias et al., 2021 | 6/8 |  |  |  |  |  |  |  |  |
| Dobson et al., 2021 | 7/8 |  |  |  |  |  |  |  |  |
| Duru, 2021 | 8/8 |  |  |  |  |  |  |  |  |
| Greenberg et al., 2021 | 8/8 |  |  |  |  |  |  |  |  |
| Hong et al., 2021 | 8/8 |  |  |  |  |  |  |  |  |
| Lamb et al., 2021 | 8/8 |  |  |  |  |  |  |  |  |
| Majumder et al., 2021 | 6/8 |  |  |  |  |  |  |  |  |
| Mediavilla et al., 2021a | 7/8 |  |  |  |  |  |  |  |  |
| Mediavilla et al., 2021b | 7/8 |  |  |  |  |  |  |  |  |
| Mortier et al., 2021 | 7/8 |  |  |  |  |  |  |  |  |
| Mosolova et al., 2021 | 7/8 |  |  |  |  |  |  |  |  |
| Murata et al., 2020 | 7/8 |  |  |  |  |  |  |  |  |
| Parthasarathy et al., 2021 | 7/8 |  |  |  |  |  |  |  |  |
| Sahimi et al., 2021 | 8/8 |  |  |  |  |  |  |  |  |
| Xu et al., 2021 | 7/8 |  |  |  |  |  |  |  |  |
| Young et al., 2021 | 8/8 |  |  |  |  |  |  |  |  |
| Mamun et al., 2020 | 6/8 |  |  |  |  |  |  |  |  |
| Rathod et al., 2020 | 7/8 |  |  |  |  |  |  |  |  |
| Xiaoming et al., 2020 | 8/8 |  |  |  |  |  |  |  |  |

Yes: , No: , Unclear or Not applicable:

**Table S2.** Overall appraisal of Case Control Studies

| **Estudio** | **JBI** | **Were the groups comparable other than presence of disease in cases or absence of disease in controls?** | **Were cases and controls appropriately matched?** | **Were the same criteria used for identification of cases and controls?** | **Was exposure measured in a standard, valid and reliable way?** | **Was exposure measured in the same way for cases and controls?** | **Were confounding factors identified?** | **Were strategies to deal with confounding factors stated?** | **Were outcomes assessed in a standard, valid and reliable way for cases and controls?** | **Was the exposure period of interest long enough to be meaningful?** | **Was appropriate statistical analysis used?** |
| --- | --- | --- | --- | --- | --- | --- | --- | --- | --- | --- | --- |
| Cai et al., 2020 | 10/10 |  |  |  |  |  |  |  |  |  |  |

Yes: , No: , Unclear or Not applicable:
